# Evaluation of the implementation of single screening and treatment for the control of malaria in pregnancy in Eastern Indonesia: a systems effectiveness analysis

**DOI:** 10.1186/s12936-018-2448-5

**Published:** 2018-08-24

**Authors:** Jayne Webster, Ansariadi, Faustina Helena Burdam, Chandra Umbu Reku Landuwulang, Jane Bruce, Jeanne Rini Poespoprodjo, Din Syafruddin, Rukhsana Ahmed, Jenny Hill

**Affiliations:** 1 Faculty of Infectious and Tropical Diseases, London School of Hygiene and Tropical Medicine, London, UK; 2 Department of Epidemiology, School of Public Health, Hasanuddin University, Makassar, Indonesia; 3 Timika Research Facility (Papuan Community and Health Development Foundation), Timika, Indonesia; 4 Eijkman Institute for Molecular Biology, Jakarta, Indonesia; 5 Paediatric Research Office, Department of Child Health, Universitas Gadjah Mada, Yogyakarta, Indonesia; 6 Mimika District Hospital, Timika, Indonesia; 7 Department of Clinical Sciences, Liverpool School of Tropical Medicine, Liverpool, UK

## Abstract

**Background:**

Indonesia introduced single screening and treatment (SST) of pregnant women for the control of malaria in pregnancy in 2012. Under this policy pregnant women are screened for malaria at their first antenatal clinic (ANC) visit and on subsequent visits are tested for malaria only if symptomatic. The implementation of this policy in two districts of Indonesia was evaluated. Cross sectional survey structured observations of the ANC visit and exit interviews with pregnant women were conducted to assess health provider compliance with SST guidelines. Systems effectiveness analysis was performed on components of the strategy. Multiple logistic regression was used to test for predictors of women being screened at their first ANC visit.

**Results:**

A total of 865 and 895 ANC visits in Mimika and West Sumba across seven and ten health facilities (plus managed health posts) respectively, were included in the study. Adherence to malaria screening at first ANC visit among pregnant women was 51.4% (95% CI 11.9, 89.2) in health facilities in Mimika (94.8% in health centres) and 24.8% (95% CI 10.3, 48.9) in West Sumba (60.0% in health centres). Reported fever was low amongst women presenting for their second and above ANC visit (2.8% in Mimika and 3.5% in West Sumba) with 89.5% and 46.2% of these women tested for malaria in Mimka and West Sumba, respectively. Cumulative systems effectiveness for SST on first visit to ANC was 7.6% for Mimika and 0.1% for West Sumba; and for second or above visits to ANC was 0.7% in Mimika and 0% in West Sumba. Being screened on a 1st visit to ANC was associated with level of health facility in both sites.

**Conclusion:**

Cumulative systems effectiveness of the SST strategy was poor in both sites. Both elements of the SST strategy, screening on first visit and passive case detection on second and above visits, was driven by the difference in implementation of malaria testing in health centres and health posts, and by low malaria transmission levels and reported fever.

## Background

Indonesia has a diverse malaria epidemiology and one of the highest burdens of malaria in Southeast Asia. The 6000 inhabited islands, with a population of approximately 230 million people, have high heterogeneity of risk of infection, malaria incidence, and Anopheles distribution [1]. Five species of *Plasmodium* are present in Indonesia. The relative proportions of *Plasmodium vivax* cases as compared to *Plasmodium falciparum* has been increasing [2] and is of concern due to high-grade multidrug-resistance to *P. vivax* in Papua Province in particular [3, 4].

Malaria in pregnancy (MiP) has devastating consequences for both the mother and the baby. In Indonesia in 2007, 6.4 million pregnancies occurred in areas with *P. falciparum* and/or *P. vivax* transmission [5]. The clinical effects of MiP depend upon the level of transmission, the malaria species and the level of immunity in pregnant women. Both *P. falciparum* and *P. vivax* contribute to the burden of MiP in Indonesia. *Plasmodium falciparum* malaria infections in pregnancy are associated with severe maternal anaemia, fetal loss and low birth weight (LBW), whilst *P. vivax* is associated with maternal anaemia, LBW and preterm births [6–8].

The Asia–Pacific region has no standardized and widely recognized strategy for prevention of MiP. In malaria endemic areas outside of sub-Saharan Africa, including the Asia–Pacific region, the strategy for MiP is the use of long-lasting insecticidal nets (LLINs) and passive case detection (PCD). In 2012 however, in malaria endemic areas Indonesia introduced single screening and treatment (SST) on first visit to antenatal clinic (ANC) followed by PCD at all subsequent visits [9], together with provision of an LLIN. The nationally recommended treatment for MiP in the 2nd and 3rd trimesters in Indonesia is dihydroartemisinin-piperaquine (DHP) and quinine in the first trimester when the study was conducted.

The heterogeneous nature of malaria epidemiology, co-existence of *P. falciparum* and *P. vivax*, together with socio-economic and cultural differences across different islands of Indonesia are likely to contribute to variations in the effectiveness of malaria control tools. In the case of SST, differences in levels of adherence to and implementation of the strategy between districts/provinces will also contribute to variations in its effectiveness. The aim of the study was to evaluate for the first time since its introduction, the implementation of SST for the control of malaria in pregnancy as per the national guidelines in two islands of Eastern Indonesia, Papua and Sumba.

## Methods

### Study site

The study was undertaken in Mimika District, Papua Province in Eastern Indonesia from February to August 2013; and in West Sumba District, Sumba, in East Nusa Tenggara Province from June to November 2014. Papua is one of the seven main islands of Indonesia and Sumba is a small island, one of the Lesser Sunda islands. Mimika District has a population of 202,359 [10], which includes a diverse number of ethnic groups mainly due to economic migration to Timika’s mining industry, and the National Transmigration Programme. West Sumba has a population of 121,901.

Malaria transmission in Papua is unstable and occurs mainly in the lowland areas, where the majority of the population live. Recent estimates of malaria prevalence in Papua for the general population are 12.2% [2] in 2016, and 16.8% for pregnant women at delivery [11], in 2008. Prevalence estimates are lower in Sumba at 6.8% in the wet season and 4.9% in the dry season in 2009 [12] in the general population, and 5.5% in pregnant women in 2005 [13].

The topography of Mimika is such that not all areas of the sub-district, and therefore health centres, are readily accessible. For example, some health centres take more than 2 days to reach by boat, and for others access requires chartering an aeroplane. Accessibility is not a problem in West Sumba with its landscape of low, limestone hills.

### Health system structure

The major structures for delivering health care are hospitals, community health centres (*puskesmas*), sub health centres (*pustus*) and community integrated village health posts (*posyandus*). Health centres, are mainly located one per sub-district (serving a population of approximately 30,000 people) and provide maternal and child care, family planning and in-patient and outpatient services. Each health centre manages several health posts, and the number of health posts managed varies between health centres. The health posts provide maternity and child health services, family planning, nutritional development, immunization and diarrhoea control. The study was conducted in hospitals, health centres and health posts. Mimika has 2 hospitals one of which is government run. There are 23 health centres, 32 sub health centres, and 129 health posts. The district of West Sumba has 2 hospitals, one of which is government run, 9 health centres, and 75 health posts.

### Study design

A mixed methods observational study was conducted using cross sectional surveys at hospitals, health centres and health posts in the two study sites at different time points. Observations and exit interviews of the ANC visit were conducted to assess compliance with national SST guidelines. The quantitative study was supplemented by in-depth interviews with health workers and focus group discussions with pregnant women accessing ANC to understand quantitative observations, these findings are reported elsewhere [14].

### The intervention

**Fig. 1:**
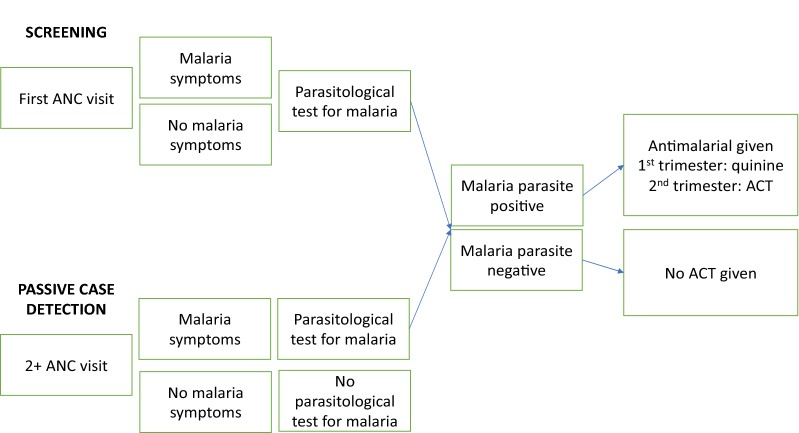
SST guideline overview

Under the SST guidelines [9], all women on their first visit to ANC should be given a parasitological test for malaria either by microscopy or RDT, regardless of symptoms, together with an LLIN (Fig. 1). On the second and subsequent visits to ANC, pregnant women are tested for malaria only if they have symptoms of suspected malaria, that is PCD. Pregnant women should be treated with quinine or an artemisinin combination therapy (ACT) DHP, depending upon their trimester, if they have a positive parasitological test. Pregnant women who have a negative parasitological test for malaria, should not be given an anti-malarial.

### Sample size

A sample size of 889 women was required for each of the study sites, based upon a conservative estimate of 50% of women reaching the SST endpoint (receiving the correct number of tablets of DHP), a design effect of 1.6 [15] and a standard error of 0.03 [16]. The sample size at each hospital and health centre was proportional to the number of 1st + 4th ANC visits in the previous year (2012 for Papua and 2013 for Sumba).

### Selection of participants and data collection

In each study site the sampling units for the cross sectional survey were the hospital and community health centres. The hospitals were purposively selected and a sampling frame of health centres constructed. Each health centre sampling unit included the health centre itself, together with the health posts under its management. In Mimika, all health centres that were logistically feasible to reach were included in the sampling frame, and in West Sumba all health centres were included. According to routine health system data, the inaccessible health centres in Mimika had very low estimated populations of pregnant women in their catchment areas and were therefore not a significant sampling loss.

In Papua and Sumba, data on number of ANC visits (1st and 4th) are manually collected in paper format at both health centres and health posts. Numbers are then collated at the health centre and submitted to the health management information system (HMIS) as total numbers for the health centre and its associated health posts. Given this health system data collection procedure, it was not possible to disaggregate data on past ANC visits by health centre and health post. ANC outreach at individual health posts was conducted once or twice per month, and sampling for the study was conducted based on the health post schedules, that is, the ones that were conducted during the time of the survey. Sampling was alternated daily between health centres and health posts, until the required sample size for each health centre and its associated health posts was reached.

### Data collection instruments and procedures

Data collection instruments included a structured questionnaire for exit interviews with ANC attendees, a structured checklist for ANC observations, and a structured health facility audit. Field workers and supervisors were trained in data collection over a period of 6 weeks in Mimika and 3 weeks in West Sumba. The head of each health facility was approached and informed about the study, and their permission to conduct the study requested. Once the signed consent of the head of the health facility was gained, then other health workers were approached, informed about the study and signed consent to be observed in their delivery of services to pregnant women accessing ANC, sometime over the following few weeks. The health facility audit was conducted by one of the fieldworker team supervisors, to collect information on numbers of women accessing ANC in the previous year, availability of microscopes and RDTs, and stocks of DHP.

Fieldworkers conducted the observations and interviews with ANC attendees by approaching a woman as she entered the health facility, they then introduced the study, gained the written consent of the woman, observed her ANC visit, and interviewed her on exit. On completion of the process with the first woman they then approached the next woman to enter the facility and repeated the process. During the ANC observations, the fieldworkers used the structured checklist to record what they saw and heard including verbal exchanges between the health worker and pregnant woman on illness and symptoms relating to malaria, conduct of a parasitological test for malaria, referral for a parasitological test, and any drugs given by directly observed therapy, or to be taken at home. The exit interview included direct questions to the pregnant woman on her demographics, pregnancy history, previous attendance at ANC, events during the just completed ANC visit, examination of any drugs received for taking at home, and knowledge on how to take the drugs. Interviews were conducted in Bahasa Indonesia, the national language of Indonesia.

### Data analysis

#### Definitions

##### Malaria test

for the purposes of this analysis, having a malaria test was defined as having an RDT conducted, or a slide prepared for malaria microscopy. Data from observations.

##### Screening

was defined as having a malaria test at a first ANC visit. Data from observations.

##### Passive case detection

a malaria test in women with suspected malaria. Data from observations.

##### Suspected malaria

suspected malaria was defined as fever reported by the health worker or the pregnant woman [17]. Data from observations.

##### Malaria symptoms

malaria symptoms were defined as headache, general pain, general weakness, flu, nausea, vomiting or dizziness. Data from observations.

Data were double entered and validated using EpiData version 3.1, Stata 14.0 was then used for data processing and analysis. Analyses accounted for the survey design, adjusting for clustering within health facilities. The cross-sectional survey was based on a single visit to ANC by a pregnant woman and the unit of analysis was therefore a woman’s ANC visit. Prevalence of malaria symptoms at first and 2nd and above (2+) visits to ANC were described. A systems effectiveness analysis [18] was undertaken comprising an assessment of the effectiveness of individual intermediate processes in SST and cumulative overall effectiveness of SST for first ANC and second and above ANC visits by pregnant women based on Fig. 1. The effectiveness of each intermediate process in the delivery system effectiveness algorithm was calculated by estimating the proportion of women who successfully reached each step from the previous step [19, 20].

Principal components analysis (PCA) was used to create an asset index [21] based upon household characteristics including drinking bottled water, having a flush toilet, having no toilet facilities, cooking on wood, having: cement floors, solar power, a radio, a television, a mobile telephone, a landline, a fridge, a bike, a motorbike, a car, and a boat. All assets were included in the PCA as binary variables [22]. The asset index was then used to construct socio-economic quintiles from the poorest households through to the least poor. In this study, these socio-economic quintiles were not representative of the population level, but are a relative score amongst women who attend ANC in each site.

Univariate (unadjusted) logistic regression was used to test for an association between being screened for malaria on 1st visit to ANC and socio-demographic, pregnancy related factors and suspected malaria, including trimester, gravidity, age, marital status, level of education, religion, level of facility at which the visit to ANC was made, socio-economic quintile, and reported fever. Any factors with an odds ratio (OR) significant at the 10% level (p-value < 0.1) were included in multi-variable (adjusted) logistic regression model to determine which potential predictors of having a malaria test on first visit to ANC, were associated when adjusted for other predictors. In the multivariable model, predictors were considered significant at the 10% level at all stages of model building except for the final model where p < 0.05 was used.

### Ethics

Consent for the study was obtained from the Institutional Review Board and Research Ethics Committees (REC) at the Eijkman Institute for Molecular Biology, Indonesia, and the Liverpool School of Tropical Medicine (LSTM). Endorsement was obtained from the Litbanges (NIH), Ministry of Health, Indonesia and deferral to the LSTM REC by the ethics committee of the London School of Hygiene and Tropical Medicine.

## Results

A total of 865 and 895 ANC visits in Mimika and West Sumba were included in the study across 7 and 10 health facilities, respectively (Table 1). In Mimika, 28.2% of the visits were to hospitals, 48.9% to health centres, and 35.9% to health posts; in Sumba, the distribution of ANC visits sampled was 17.1% to hospitals, 39.2% to health centres and 60.8% to health posts. The sampling distribution in Mimika, based on proportions of 1st and 4th visits during the previous year, was highly focussed in two health centres, with one of these and its managed health posts comprising 36.1% of the overall sample. In West Sumba, except for one health centre and its health posts that formed 29.2% of the overall sample, ANC visits sampled across health facilities were more evenly spread.

**Table 1:** Sampled ANC attendees in Mimika and West Sumba

Mimika					West Sumba				
Facility	Hospital	Health centre	Health post	Total	Facility	Hospital	Health centre	Health post	Total
Mimka 1	216			216	Sumba 1	153			153
Mimka 2		192	184	376	Sumba 2		2	62	64
Mimika 3		149	5	154	Sumba 3		5	42	47
Mimika 4		28	0	28	Sumba 4		44	217	261
Mimika 5		4	52	56	Sumba 5		29	24	53
Mimika 6		0	34	34	Sumba 6		61	34	95
Mimka 7		1	0	1	Sumba 7		10	39	49
					Sumba 8		37	2	39
					Sumba 9		8	80	88
					Sumba 10		2	44	46
Total	216	374	275	865		153	351	544	895

Health centres in the districts studied in both sites were managing a median of 10 and 12 health posts, ranging from 5 to 22 in Mimika and 2 to 19 in West Sumba. In the year previous to the surveys (2012 for Mimika and 2013 for West Sumba), numbers of new ANC clients were higher in hospitals of Mimika than in West Sumba (1280 and 286, respectively), median numbers for health centres and their managed health posts were similar, but with a skew towards two larger health centres in Mimika. Four health centres had a medical doctor in Mimika, whereas none of the health centres had medical doctors in West Sumba. Numbers of midwives varied from 13 in the larger health centre in Mimika to none in a relatively small health centre in West Sumba. All except 1 health centre in Mimika had 1–2 functioning microscopes. Only 2 of the 6 (33%) health centres in Mimika had received RDTs in the previous 6 months (prior to the study date), had RDTs at the time of the survey and reported that they used RDTs in ANC. In West Sumba, 2 of the 9 (22%) health centres had received RDTs in the previous 6 months and had them at the time of the survey; none of the health centres reported using RDTs in ANC. Equipment for ANC, including RDTs when available, was taken on outreach days from the health centres to the health posts, and therefore at the time of the study lack of RDTs in the health centres meant that the majority of health posts in both sites did not have access to RDTs. In Mimika, 2 health centres reported that their health posts perform RDTs, 2 that their health posts make slides and send them to the health centre and 2 that their health posts refer women elsewhere for malaria tests. In West Sumba, none of the health centres reported that their health posts perform RDTs, one health centre said that their health posts make slides and send to the health centre, and others that they refer women elsewhere for malaria parasite tests.

The characteristics of pregnant women sampled in both sites were similar, approximately 50% were in the second trimester, 50% had already given birth twice or more, approximately 60% were 20–29 years of age, and the majority were married (Table 2). Numbers of visits to ANC were high, with the current visit for nearly 50% of women in both sites being their 4th or more visit.

**Table 2:** Characteristics of sampled ANC attendees in Mimika and West Sumba

	Mimika			West Sumba		
	N	%	95% CI	N	%	95% CI
Trimester						
1st trimester	138	16.0	12.8, 19.8	140	15.7	12.5, 19.5
2nd trimester	436	50.5	36.4, 64.4	485	54.3	44.9, 63.2
3rd trimester	290	33.6	19.6, 51.1	268	30.0	20.8, 41.3
Gravidity						
Gravida 1	249	28.8	24.6, 33.5	270	30.4	24.9, 36.5
Gravida 2	256	29.6	26.5, 33.0	207	23.3	19.8, 27.2
Gravida ≥ 3	359	41.6	35.7, 47.7	411	46.3	42.2, 50.5
Age group						
12–19	70	8.1	6.3, 10.4	57	6.4	4.0, 10.1
20–29	536	62.0	54.6, 68.8	507	56.7	50.6, 62.6
30–39	242	28.0	20.7, 36.7	292	32.6	28.1, 37.5
≥ 40	17	2.0	1.0, 3.7	39	4.4	2.7, 7.1
Marital status						
Single	141	16.3	13.9, 19.0	48	5.4	4.1, 7.0
Married	722	83.5	80.9, 85.7	845	94.4	92.6, 95.8
Divorced	2	0.2	0.0, 1.8	2	0.2	0.1, 0.8
Widowed	0	0	0	0	0	0
Education						
Not attended school	39	4.5	1.2, 15.5	87	9.7	4.9, 18.5
None completed	10	1.2	0.4, 3.4	89	9.9	6.9, 14.2
Primary	120	13.9	10.2, 18.6	232	25.9	17.6, 36.4
Secondary	189	21.9	18.0, 26.4	162	18.1	14.5, 22.4
Post-secondary/technical	419	48.5	43.1, 53.9	249	27.8	22.0, 34.5
Tertiary	87	10.1	6.6, 15.0	76	8.5	2.3, 26.7
Religion						
Catholic	171	19.8	16.2, 23.9	166	20.9	15.4, 27.7
Protestant	370	42.8	32.1, 54.2	610	76.7	69.8, 82.5
Muslim	324	37.5	28.9, 46.9	19	2.4	0.7, 8.1
ANC Visit number						
1	185	21.4	13.4	161	18.0	12.7, 24.8
2	145	16.8	14.1, 19.9	169	18.9	14.9, 23.7
3	132	15.3	12.8, 18.2	138	15.4	13.1, 18.0
4+	403	46.6	33.7, 60.0	427	47.7	38.1, 57.5
First ANC visit						
Hospital	29	13.4	–	9	5.9	–
Health centre	77	20.5	12.3, 32.3	50	25.3	18.2, 33.9
Health post	79	28.8	18.2, 42.4	102	18.8	14.5, 23.9

Prevalence of fever was similar across both study sites at 3.2% (95% CI 1.1, 9.1) in Mimika and 2.8% (95% CI 1.1, 6.7) in West Sumba (Table 3). Prevalence of other potential symptoms of malaria was also similar across study sites with nausea, vomiting and dizziness being the most prevalent. Nausea, vomiting and dizziness were more prevalent in women on their first visit to ANC than on 2+ visits.

**Table 3:** Prevalence of malaria symptoms in Mimika and West Sumba on 1st and 2nd or above visit to ANC

Symptom	Mimika prevalence						West Sumba prevalence					
	1st ANC			2+ ANC			1st ANC			2+ ANC		
	n	%	95% CI	n	%	95% CI	n	%	95% CI	n	%	95% CI
Fever	6	3.2	1.1, 9.1	19	2.8	1.1, 6.7	7	4.3	1.6, 7.4	26	3.5	1.7, 7.4
Headache	7	3.8	2.0, 7.1	15	2.2	1.3, 3.6	7	4.3	1.6, 11.5	38	5.2	2.8, 9.3
General pain	9	4.9	2.2, 10.2	49	7.2	4.4, 10.2	11	6.8	3.9, 11.8	76	10.4	7.7, 13.8
General weakness	4	2.2	0.7, 6.2	4	0.6	0.3, 1.3	14	8.7	4.2, 17.1	27	3.7	1.8, 7.5
Flu	2	1.1	0.2, 6.0	15	2.2	1.3, 3.7	6	3.7	1.5, 9.1	25	3.4	1.6, 7.2
Nausea	44	23.8	17.4, 31.6	42	6.2	4.1,9.2	37	23.0	15.9, 32.1	52	7.1	5.5, 9.2
Vomiting	26	14.1	11.1,17.7	28	4.1	1.9, 8.5	23	14.3	7.2, 26.4	24	3.3	1.7, 6.0
Dizzy	28	15.1	10.4, 21.5	48	7.1	5.5, 9.1	46	28.6	21.7, 36.7	114	15.5	10.9, 21.6

### SST intermediate systems effectiveness at first ANC visit

A total of 346 first ANC visits were observed, 185 in Mimika and 161 in West Sumba. Amongst the women attending hospitals for ANC only small proportion were first visits, 13.4% in Mimika and 5.9% in West Sumba. First visits formed a higher proportion of overall visits in health posts in Mimika 28.8% (95% CI 18.2, 42.2) and health centres 25.3% (95% CI 18.2, 33.9) in West Sumba.

Malaria screening at first ANC visit among pregnant women was 51.4% (95% CI 11.9, 89.2) in health facilities in Mimika and 24.8% (95% CI 10.3, 48.9) in West Sumba (Table 4). Adherence to first ANC visit screening varied by level of health facility and was high at 94.8% (95% CI 81.1, 98.7) in health centres of Mimika (Table 5). Two-thirds of first ANC visits were screened in the hospital in Mimika compared with 0% in West Sumba. Implementation of screening for malaria in health posts of both sites was poor, with less than 10% of first ANC visits screened. Most screening conducted at first ANC visit in both sites was by microscopy. In Mimika, 1.1% (2/185) first ANC visits were screened by RDT, and in West Sumba 1.2% (2/161). All four RDTs conducted on first ANC visit were performed at health posts.

**Table 4:** Individual and cumulative systems effectiveness for SST in Mimika and West Sumba

	Mimika			West Sumba		
	n	Process effectiveness	Cumulative effectiveness	n	Process effectiveness	Cumulative effectiveness
1st ANC visit	185	100	100	161	100	100
Screened for malaria	95	51.4	51.4	40	24.8	24.8
Positive	18	18.9	9.7	1	2.5	0.1
Positive given ACT or quinine	14	77.8	7.6	1	100	0.1
≥ 2nd ACT visit	680	100	100	734	100	100
Reported fever	19	2.8	2.8	26	3.5	3.5
Screened for malaria if reported fever	17	89.5	2.5	12	46.2	1.6
Positive	7	41.2	1.0	0	0	0
Positive given ACT	5	71.4	0.7	0	0	0

**Table 5:** Malaria parasite screening at 1st ANC visit by level of health facility

	Mimika			West Sumba		
	n	%	95% CI	n	%	95% CI
Hospital	19	65.5	–	0	0	
Health centre	73	94.8	81.1, 98.7	30	60.0	32.6, 82.3
Health post	3	3.8	1.6, 8.8	10	9.8	4.4, 20.5

In Mimika, 18/95 (3.5%) first ANC visits that were screened had a positive malaria test, 2 at the hospital, 15 at a health centre, and 1 at a health post. This equates to a positivity rate of 18.9%. Amongst those first ANC visits testing positive, 11 (61.0%) were given DHP, 10 of the women given DHP were in the second trimester of their pregnancy and one was in the first trimester. Of the five women positive for malaria was given quinine, four were in their first trimester of pregnancy and one was in the second trimester. In West Sumba, only one woman on her first ANC visit tested positive for malaria (by microscopy) in a health centre. The pregnant woman was symptomatic for malaria and was given DHP. Therefore, the intermediate process effectiveness for those screened for malaria, i.e., those positive were given DHP or quinine as appropriate for their trimester, and those that were negative were not given DHP or quinine, was 92.6%. Adherence to treatment guidelines based on malaria test results was high.

No women on their first visit to ANC in Mimika or West Sumba, who were either screened negative for malaria parasites or not tested, were given DHP.

### SST intermediate process effectiveness at second or above ANC visits

A total of 1414 women on their second or above visits to ANC were sampled, 680 in Mimika and 734 in West Sumba. Amongst women attending hospitals for ANC a high proportion were second or above visits: 86.6% in Mimika and 94% in West Sumba. Second or above visits formed approximately 70% to 80% of all ANC visits to health centres and health posts in Mimika and West Sumba, respectively.

Reported fever was low amongst pregnant women on their second or above visit to ANC, 2.8% (95% CI 1.1, 6.7) in Mimika and 3.5% (95% CI 1.7, 7.4) in West Sumba. Of those women on their second or above visit to ANC with reported fever, 89.5% (95% CI 59.7, 98.0) in Mimika and 46.2% (95% CI 15.7, 79.8) in West Sumba were tested for malaria.

In Mimika amongst pregnant women on their second or above visit to ANC who had suspected malaria and were tested, 7 tested malaria positive, and 10 tested negative. Of those positive for malaria, 5 were given DHP and 1 was given quinine and amongst those negative, none were given DHP or quinine. This translates to 85.7% of women tested for malaria on a second or above visit to ANC being treated as per the national guidelines. In West Sumba none of the 12 women with reported fever who were screened for malaria were positive and none were given DHP or quinine.

### Cumulative systems effectiveness

**Fig. 2:**
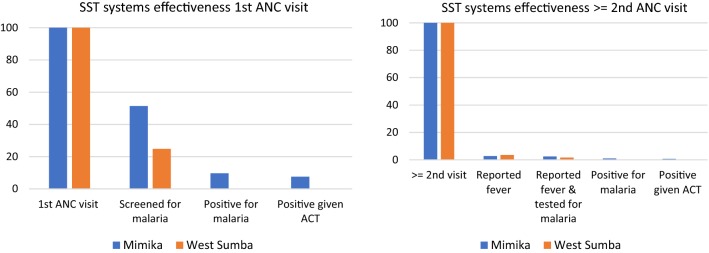
SST cumulative systems effectiveness for 1st (malaria screening) and ≥ 2nd visits (passive case detection) to ANC in Mimika and West Sumba

Cumulative systems effectiveness for SST on first visit to ANC was 7.6% for Mimika and 0.1% for West Sumba (Fig. 2); and for second or above visits to ANC was 0.7% in Mimika and 0% in West Sumba.

### Predictors of being screened for malaria on first visit to ANC

In Mimika, there was no association in univariate analyses between having a malaria test on first visit to ANC and any of the tested socio-demographic factors, pregnancy related factors, or reported fever (Table 6). Level of health facility was the only predictor of being screened for malaria on a 1st visit to ANC: reference: health centres OR 1.0; health post OR 0.002 (95% CI 0.0004, 0.01; hospital OR 0.1 (95% CI 0.02, 0.4); p = 0.0001 (Table 5). In West Sumba, predictors of having a malaria test at 1st visit to ANC in the univariate analyses included trimester, age, level of education, level of health facility and reported fever, but in the multi-variable model the only predictor was level of facility AOR 0.2 (95% CI 0.03, 0.8) p = 0.03; with reported fever dropped from the model because reported fever perfectly predicted having a malaria test.

**Table 6:** Predictors of being screened for malaria parasites at 1st ANC visit

	Mimika			West Sumba				p
	Unadjusted			n	Unadjusted	p	Adjusted	
	n	OR (95% CI)	p		OR (95% CI)		AOR (95% CI)	
Trimester								
1st	37	1.0	0.2	23	1.0	*0.02*	1.0	0.5
2nd	54	1.2 (0.7, 2.3)		15	0.5 (0.4, 0.8)		0.7 (0.3, 1.9)	
3rd	3	0.4 (0.1, 6.2)		1	0.6 (0.1, 3.5)		1.0	
Gravidity								
0	32	1.0	0.2	14	1.0	0.1		
1	26	1.1 (0.6, 2.0)		11	0.5 (0.2, 1.6)			
≥ 2	37	0.6 (0.4, 1.1)		15	0.3 (0.1, 0.9)			
Age								
12–19	12	1.0	0.2	7	1.0	*0.002*	1.0	0.9
20–29	58	0.8 (0.4, 1.5)		25	0.4 (0.1, 1.6)		0.9 (0.3, 2.7)	
30–39	22	0.5 (0.2, 1.0)		6	0.1 (0.05, 0.4)		0.7 (0.2, 2.9)	
40+	3	–		2	0.4 (0.04, 3.8)		1.0	
Marital status								
Single	23	1.0	0.3	2	1.0	0.7		
Married	72	0.7 (0.3, 1.6)		38	1.3 (0.3,7.2)			
Widowed	0	0		0	0			
Education								
None	4	1.0	0.2	5	1.0	*0.001*	1.0	0.9
None completed	1	0.3 (0.01, 1.4)		1	0.2 (0.01,2.2)		–	
Primary	16	0.8 (0.01, 57.9)		12	1.0 (0.2, 5.5)		0.7 (0.2, 2.1)	
Secondary	17	0.3 (0.01, 17.1)		10	1.7 (0.3, 9.6)		1.0 (0.2, 2.3)	
Post-secondary	49	0.6 (0.01, 30.5)		11	2.1 (0.5, 8.4)		1.6 (0.3, 8.6)	
Tertiary	8	0.7 (0.02, 20.1)		1	0.7 (0.03,13.5)		1.2 (0.006, 239.9)	
Religion								
Catholic	15	1.0	0.3	6	1.0	0.5		
Protestant	47	2.2 (0.8, 6.3)		30	1.5 (0.4, 5.3)			
Islam	33	1.5 (0.6, 3.6)		0	–			
Level of facility								
Health centre		1.0	*0.0001*	30	1.0	*0.002*	1.0	*0.03*
Health post	3	0.002 (0.0004, 0.01)		10	0.07 (0.02, 0.3)		0.2 (0.03,0.8)	
Hospital	19	0.1 (0.02, 0.4)		0	–		–	
Socio-economic quintile								
Poorest	18	1.0	0.5	5	1.0	0.1		
Very poor	20	1.5 (0.3, 6.9)		10	1.6 (0.3, 8.8)			
Poor	18	1.3 (0.3, 5.8)		12	2.7 (0.6, 11.6)			
Less poor	23	1.5 (0.4, 6.3)		9	2.7 (0.5, 16.5)			
Least poor	16	2.1 (0.5, 9.1)		4	2.3 (0.1, 38.8)			
Reported fever								
No	41	1.0	0.2	30	1.0	*0.05*	–*	
Yes	5	5.0 (0.3, 96.5)		6	11.4 (1.1, 121.5)		–*	

Italic values indicate significance of p value (*OR* p < 0.1; *AOR* p < 0.05)

* Reported fever perfectly predicted having a malaria test

## Discussion

In this study, the implementation of SST for malaria in pregnant women per national guidelines in two districts of Indonesia with different levels of malaria transmission was evaluated. Indonesia has a policy of SST for malaria in pregnancy in all areas of malaria transmission, and is one of the few countries in the Asia–Pacific Region with a specific malaria in pregnancy control policy, in addition to the distribution of LLINs. The evaluation in Mimika was approximately 2 years post policy adoption by the Ministry of Health, and in West Sumba approximately 3 years post policy.

Systems effectiveness was used to assess the proportion of pregnant women accessing ANC for first or second and above visits who received overall malaria prevention as defined by the national policy of SST, and who completed the individual intermediate processes comprising SST. Systems effectiveness has been used to assess implementation of malaria control interventions including case management, delivery of intermittent preventive treatment in pregnancy and delivery of long lasting insecticidal nets (LLINs) [18, 19, 23]. No studies of systems effectiveness for evaluating implementation of screening and treatment of malaria control interventions were available for comparison of outcomes.

Being screened for malaria on the first visit to ANC was associated with level of facility in Mimika and West Sumba, and with reported fever in West Sumba. In both study sites adherence to malaria screening on first visit to ANC were more likely to take place if the woman attended a health centre, testing was unlikely at health posts in both sites and was not given to any of the women sampled at the hospital in Sumba. The lack of association between having a malaria test and reported fever, together with the high proportion of women having a malaria test when attending health centres of Mimika, represents adherence to this element of the strategy. This was not the case in Sumba where only a small proportion of women were given a malaria test on their first ANC visit and this was associated with reported fever. As there are no published studies of this strategy, it is not possible to draw comparisons on its implementation in other countries.

Screening for malaria parasites was implemented only where microscopy was available. The use of RDTs for screening for malaria in pregnant women was not implemented in either study site, including at health posts where microscopy was not available. During some ANC visits at health posts, pregnant women had blood taken and a microscope slide prepared, but this was not common practice. It is not possible to draw on routine data to estimate the proportion of first ANC visits that take place in health posts relative to other levels of health facility, because health centre and health post data are merged before inclusion in the health management information system. However, in this study amongst those attending health posts 29% were first ANC visit in Mimika, and 19% in West Sumba. It is therefore important that either RDTs are deployed and used in health posts, or slides for malaria microscopy are taken from all first ANC visit women attending health posts. The second of these options is potentially less likely to be successfully implemented as it would require pregnant women to then attend the health centre for their results, and anti-malarials as required.

In Mimika, approximately two-thirds (64%) of first ANC visit women screened positive for malaria did not report fever, nor was fever suspected by the health worker. In the absence of screening, these women would not have been diagnosed for malaria. Sub-microscopic infections and asymptomatic infections have previously been identified in Papua [2, 24], yet the associated clinical outcomes of these infection are not well understood. In an observational study of ANC visits in eastern India, blood tests were typically obtained if a patient complained of fever, though enquiries into presence of fever in patients were made in only a minority of patients [25]. In Mimika, based on the study findings of malaria parasite prevalence compared to the prevalence of fever, parasite positive women would be missed if tested only based on the presence of symptoms.

Adherence to guidelines on treating malaria positive women with the nationally recommended drugs was high. In Mimika approximately 90% of first ANC visit women testing positive for malaria were treated with DHP or quinine. The drug they were given was associated with trimester, with the majority of first trimester women being given quinine and second trimester DHP. There were no cases of pregnant women being given a course of treatment with DHP without being tested, and found positive.

The malaria parasite prevalence in 1st visit ANC attendees in Mimika at 18.9% was slightly higher than that reported in 2008 for women at delivery in the same study site [11]. In West Sumba, it was not possible to estimate malaria parasite prevalence, as having a malaria test was associated with being febrile and therefore a case of suspected malaria. Prevalence of reported fever was lower than parasite prevalence in Mimika, that is malaria was asymptomatic in some pregnant women, and was similar in both sites at approximately 3% amongst 1st and second and above visit ANC attendees. Reported fever was not validated by recording temperature.

Passive case detection requires that women with suspected malaria are tested for malaria parasites and treated with DHP if positive. Clinical symptoms of malaria in pregnancy beyond fever, are very ill-defined, but are known to vary depending upon level of malaria transmission and the woman’s immunological response. Whilst the WHO recommendation is that any woman with a fever be treated as a suspected malaria case, it is likely that this overestimates the number of cases, as has been shown in sub-Saharan Africa [26]. There is a dearth of data on implementation of PCD for malaria in pregnancy in terms of clinical signs and symptoms that prompt health workers to suspect malaria, and request a parasitological test, and further the clinical symptoms that predict a positive parasitological test. Based on the association between symptoms and performing a malaria test in Mimika on 2+ visits, health workers view fever, vomiting and dizziness as potential symptoms of malaria, and in Sumba fever and general pain.

Despite the relative effectiveness of some individual processes in delivery of SST to first, and second and above, ANC visit pregnant women, the cumulative effectiveness was low. The cumulative effectiveness starting point was the visit to ANC and the endpoint was the receipt of DHP or quinine. In this study, the cumulative effectiveness was dependent upon the decisions made by ANC health workers, whether to screen on first visit or test on second and above, and their access to malaria tests; but also on malaria transmission, being positive for malaria when screened, and being reported with suspected malaria, i.e., febrile on a second or above visit to ANC.

Systems effectiveness analyses in malaria control have varied in their starting points including accessing ANC [19, 23], being pregnant [27], and people or children with current or recent fever [27], and in the processes included. For some, all those included at step one should be eligible for each subsequent process as for example in the delivery of IPTp [23], whereas others including case management will only be eligible for treatment depending upon a positive malaria test [28].

The study was undertaken pre-implementation of a cluster randomized controlled superiority trial of the current policy of SSTp-DHP versus two alternative strategies which were intermittent screening and treatment (ISTp-DHP) and IPTp-DHP. The trial hypothesis was that among pregnant women protected with LLINs, intermittent screening with RDTs at ANC visits provided monthly during pregnancy and treatment of RDT positive women with DHP (ISTp-DHP), or IPTp with DHP (IPTp-DHP) is more efficacious than SST (SSTp-DHP) in preventing malaria in pregnancy in an area of relatively low prevalence of *P. falciparum* and *P. vivax*.

Findings of this current observational study supplement the trial findings in taking forward a strategy for malaria prevention in Eastern Indonesia. For either SSTp-DHP or ISTp-DHP to be effectively implemented, malaria tests and drugs need to be introduced and sustained at all health posts either by RDTs or by taking a microscope slide for malaria to be tested at the health centre. The use of RDTs would be preferable as this is more likely to result in the woman being tested and treated, than the relatively complex system of ensuring that the result is received by the woman and treatment dispensed appropriately. A cost effectiveness study would be an important next step, but given the low level of systems effectiveness it is unlikely that SST would be cost effective in this setting.

Given the level of sub-microscopic malaria it is logical to hypothesize that IPTp-DHP would be superior to ISTp-DHP in Mimika. Should this be the case, much work will be needed to convince health workers, particularly in health centres, that malaria tests are not needed when they are currently adhering so well to screening at first ANC visit. Conversely, in health posts, where malaria testing is not currently implemented, the acceptance of IPTp-DHP is likely to be relatively straight forward. The adherence to the SSTp-DHP strategy may however indicate a general propensity to adhere to guidelines and, therefore, implementation of IPTp-DHP too, may be potentially successful. The qualitative study conducted alongside this evaluation explores further the perceptions of health workers on these strategies [14].

The study design had several limitations. Longitudinal following of women through their pregnancy to capture all visits to ANC was not included and the findings are therefore only a snapshot of individual visits to ANC. Due to the inaccessibility of some areas of the sub-district and its health facilities in Mimika it was not possible to select samples from a sampling frame of all health centres. The unavailability of data distinguishing ANC visits at health centres versus health posts meant that it also was not possible to select samples stratified by these levels of health facility. The process for enrolling pregnant women at each health facility was based upon implementation feasibility and was not technically random. There may have been a bias towards women attending at particular times of day and being given a parasitological test, however in Mimika and West Sumba approximately 95% and 93%, of women, respectively, began their ANC consultation before 10 a.m.

Non-participant structured observations together with exit interviews were the main tool for assessing the implementation of SST. It is possible that the health workers and the pregnant women changed their behaviours because they were being observed, this phenomenon is called the Hawthorne effect [29, 30]. It was assumed that the participants exhibited their best behaviours at the time of being observed. It has been shown, that participants tend to revert to their normal practices after a small number of observations [30]. The behaviour of health workers was, therefore, likely to be normalized during the study, but not that of pregnant women.

## Conclusion

Adherence to guidelines on SST for malaria prevention differed across the components of the strategy, across study sites, and by level of health facility within both Mimka and West Sumba. Cumulative effectiveness of SST for women on their first visit and women on their second or above visits was low. Screening and testing was almost exclusively by microscopy. In Mimika, the high proportion of women screened positive for malaria who were asymptomatic means that passive case detection is not effective in detecting malaria in this setting. Generally, the treatment component of SST was adhered to with malaria positive women in first trimester given quinine, second/third trimester given DHP, and women not tested positive for malaria not treated.
